# KM-express: an integrated online patient survival and gene expression analysis tool for the identification and functional characterization of prognostic markers in breast and prostate cancers

**DOI:** 10.1093/database/bay069

**Published:** 2018-07-09

**Authors:** Xin Chen, Zhengqiang Miao, Mayur Divate, Zuxianglan Zhao, Edwin Cheung

**Affiliations:** 1 Guangdong Key Laboratory of IoT Information Technology, School of Automation, Guangdong University of Technology, No. 100 Waihuan Xi Road, Guangzhou Higher Education Mega Center, Panyu District, Guangzhou 510006, PR China; 2 Faculty of Health Sciences (E12), University of Macau, Avenida da Universidade, Room 4045, Taipa, Macau, China

## Abstract

The identification and functional characterization of novel biomarkers in cancer requires survival analysis and gene expression analysis of both patient samples and cell line models. To help facilitate this process, we have developed KM-Express. KM-Express holds an extensive manually curated transcriptomic profile of 45 different datasets for prostate and breast cancer with phenotype and pathoclinical information, spanning from clinical samples to cell lines. KM-Express also contains The Cancer Genome Atlas datasets for 30 other cancer types with matching cell line expression data for 23 of them. We present KM-Express as a hypothesis generation tool for researchers to identify potential new prognostic RNA biomarkers as well as targets for further downstream functional cell-based studies. Specifically, KM-Express allows users to compare the expression level of genes in different groups of patients based on molecular, genetic, clinical and pathological status. Moreover, KM-Express aids the design of biological experiments based on the expression profile of the genes in different cell lines. Thus, KM-Express provides a one-stop analysis from bench work to clinical prospects. We have used this tool to successfully evaluate the prognostic potential of previously published biomarkers for prostate cancer and breast cancer. We believe KM-Express will accelerate the translation of biomedical research from bench to bed.

Database URL: http://ec2-52-201-246-161.compute-1.amazonaws.com/kmexpress/index.php

## Introduction

The most commonly diagnosed cancers in men and women are prostate cancer and breast cancer, respectively (1). For these cancers, hormone-deprivation therapies are used with or without surgery as first-line treatments (2, 3). Unfortunately, these cancers often demonstrate either *de novo* resistance to hormonal therapies or subsequently acquire resistance following an initial therapeutic response (3). Thus, it is important to identify prognostic markers for disease progression and resistance to treatments, and to predict the patients’ outcome. Comprehensive functional analysis is also needed to accelerate the clinical use of these markers as therapeutic targets.

To aid researchers in identifying new prognostic markers, a number of gene expression and survival analysis web-tools for patient data analysis have been developed. For example, web-tools such as Oncomine (4, 5), GENT (6), BioXpress (7) and MERAV (8) can be used for the mining of patient gene expression data. In general, these web-tools allow cross-dataset expression analysis between normal and disease states, during the progression of the disease, or in response to treatment, and thus are useful for confirming prognostic markers. For patient survival analysis, a number of options are also available and they include but not limited to PrognoScan (9), G-DOC (10), GOBO (11), SurvExpress (12), BreastMark (13), SurvMicro (14) and Kaplan–Meier Plotter (15–19). Currently, most of these web-tools house only microarray datasets but with the recent availability of next-generation sequencing (NGS) data from projects such as The Cancer Genome Atlas (TCGA), web-tools such as The cBio Cancer Genomics Portal (http://cbioportal.org) (20, 21), SurvExpress (12) and PROGgeneV2 (22) are beginning to include these datasets as well.

Besides discovering new prognostic markers for cancers, it is also important to characterize their biological functions, since they could also be potential therapeutic targets. However, in order to examine the function of biomarkers, it is necessary to find a cell model system that expresses the genes of interest in order to mimic as close to the patient as possible. For example, to study estrogen receptor (ER)-positive breast cancer most researchers will choose the model cell lines MCF7 or T47D which over-expresses ER. As of now, CellMinerHCC (23), CellLineNavigator (24), GENT (6), COSMIC (25) and MERAV (8) are web-tools that can be used for cell line gene expression analysis. CellMinerHCC is a database containing gene expression data of 18 hepatocellular carcinoma (HCC) cell lines, whereas CellLineNavigator, COSMIC and MERAV contain gene expression profiles of diverse cancer cell lines based on microarray profiles and different pathological states and tissues.

Although there are many web-tools available to aid researchers in biomarker discovery, it can still be a confusing and daunting task for them as they need to search and determine which tools are best suited for each of the specific analysis as described above. Thus, it would be useful and much easier if there exists a single tool containing the latest datasets that researchers can use to perform all of the tasks for biomarker discovery as well as functional characterization. However, to the best of our knowledge, there is currently no web-tool that contains all the necessary analysis and information in one single package. To this end, we have constructed KM-Express, a simple and easy to use web-tool that combines, (i) patient survival data, (ii) patient gene expression analysis and (iii) cell line gene expression information. KM-Express holds 36 manually curated RNA-seq data for prostate and breast cancers with 7 cell line datasets from public databases. The current version of KM-Express also includes TCGA datasets for 30 other cancer types along with cell line data support for 23 of them. In summary, KM-Express simultaneously provides survival analysis, cross-dataset and subgroup expression comparison, statistical test, experimental support from cell line and visualization of the data. We present this tool as a hypothesis generation tool to help researchers to identify potential prognostic and diagnosis biomarkers. Finally, this web-tool provides a one-stop analysis platform from bench work to clinical prospects and will greatly facilitate the process of biomarker discovery.

## Materials and methods

### Data acquisition

We searched in PubMed (http://www.ncbi.nlm.nih.gov/pubmed) for publications with keywords related to breast/prostate and RNA-seq. Specifically, we used the following search terms: ‘prostate AND cancer AND ((RNA AND sequencing) OR RNA-seq)’ for prostate cancer, and ‘breast AND cancer AND ((RNA AND sequencing) OR RNA-seq)’ for breast cancer. A biologist might be interested in specific questions related to drug treatment, the overexpression, or knock-down of genes in cell lines. In the current version of KM-Express, we have also included such datasets. From publications, we have obtained the accession ID and downloaded data from the Short Reads Archive (http://www.ncbi.nlm.nih.gov/sra) or dbGaP (http://www.ncbi.nlm.nih.gov/gap) for the authorized dataset (phs000310.v1.p1). Clinical data were sorted from series matrix file(s) on Gene Expression Omnibus (GEO) (http://www.ncbi.nlm.nih.gov/geo/). Gene expression datasets from TCGA was downloaded from https://gdc-portal.nci.nih.gov/projects/t. RNA-seq FPKM data were log2 transformed when comparing the expression level of the different subgroups. The accession IDs and data descriptions are shown in the web interface.

### Processing of RNA-Seq data

SRA files were converted into fastq format using fastq-dump from the SRA Toolkit (https://trace.ncbi.nlm.nih.gov/Traces/sra/sra.cgi? view=software). The reads from each library were aligned uniquely and independently with TopHat (version 2.0.6) (26) to the hg19 human reference genome which is available at http://bowtie-bio.sourceforge.net/bowtie2/index.shtml. All of the parameters used were default except for: –splice-mismatches 1, –mate-inner-dist 200, –library-type fr-unstranded and –transcriptome-index. Based on the mapped reads, FPKM values for transcripts were calculated using Cufflinks (version 2.0.2) (26) with default parameters except for –max-bundle-frags 500 000 and –multi-read-correct. Genes were discarded if they were not expressed at appreciable levels (FPKM > 1) in at least half of all the samples in the selected dataset.

### Webpage design

**Figure 1.**
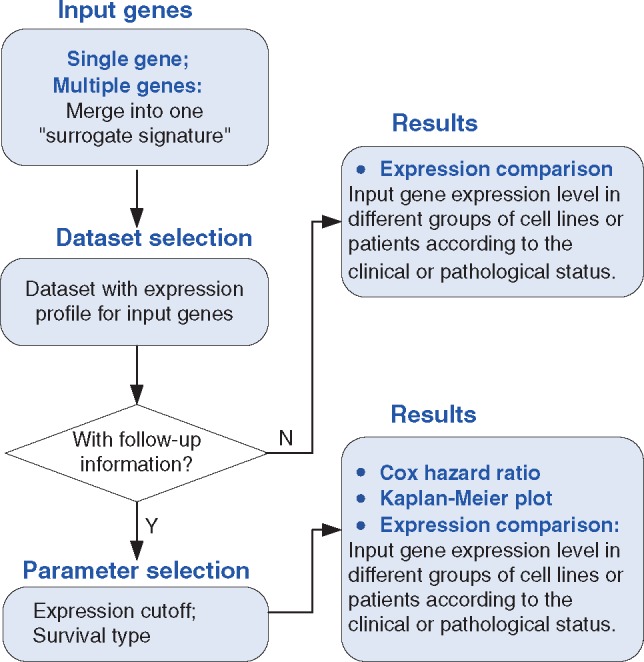
Overview of the KM-Express web-tool. A flowchart showing how KM-Express works. If the input genes are expressed in the selected dataset, KM-Express will generate an output of their expression level in different groups of patients or cell lines according to the clinical or pathological parameters. If the selected dataset contains follow-up information, the user will be asked to select the expression cutoff to define the high and low expressed groups of patients. After the user chooses the available patient outcome, logrank test is used to determine if high and low expressed groups of patients have significantly different survival rates. The output will contain the Kaplan–Meier plot with hazard ratio and significance *P*-value. If the input contains multiple genes, the survival analysis will be done for the surrogate gene, which represents the average expression level of all the input genes.

A summary of the webpage design for KM-Express is shown in Figure 1. Specifically, the webpage consists of four parts: ‘Input genes’, ‘Dataset Selection’, ‘Parameter Selection’ and ‘Results’. If the input genes are expressed in the dataset, then the output of their expression levels in the different groups of patients and cell lines will be shown according to the clinical or pathological status. If the selected dataset also contains follow-up information, KM-Express will perform survival analysis on this dataset. Users can select the expression level cutoff to define the high and low expressed groups of patients, choose the available patient outcome (alive or dead; recurrence or not), and check if patients with high and low expression of input genes have a significant difference in outcome. The output of the results will show the Kaplan–Meier plot containing the hazard ratio and *P*-value.

The KM-Express web-tool was constructed using PHP and JavaScript. Survival and gene expression analyses were performed using R. PHP was used to pass the user-selected parameters from the webpage to the R program and to visualize all the output results and figures. To increase the speed of data processing by R, RData was used as the format for dataset storage, which can be fast when loading large expression profiles into R. Additionally, the expression datasets were processed and stored in the unified RData format, and thus there is no need to revise the scripts even when new datasets are added in the future. Therefore, RData formatted expression profiling makes KM-Express run faster and easier to maintain.

We have included a function panel called ‘DataSubmit’ that allows users or maintainers of KM-Express to easily update the datasets. Users simply submit two files, one for the gene expression profiling and the other for the sample annotation with or without available phenotype information. Examples of submitted files are provided. After being checked and curated by the administrator, the submitted files will be loaded and saved as RData format for survival and expression analysis in KM-Express. We will update the datasets for KM-Express every 6 months.

### Input gene(s)

The official gene symbol from the HUGO Gene Nomenclature Committee (HGNC) is the only acceptable ID for entering genes in KM-Express. If entering multiple genes, the survival analysis will be performed for the surrogate gene, which represents the average expression level of all input genes. The expression analysis for patient samples or cell lines will show output expression comparison for each input gene. KM-Express will not process any genes with a low expression level (FPKM < 1 in more than half of the samples in the selected dataset).

### Hazard ratio and *P*-value

The R package ‘survival’ was used to calculate and plot Kaplan–Meier survival curves (27, 28).

## Results

### Data source

Currently, KM-Express contains extensive transcriptomic expression profiling based on RNA-seq data for prostate and breast cancers. Specifically, we have manually curated 13 datasets for prostate cancer and 25 datasets for breast cancer from various publications (Table 1 and Supplementary Table S1). In addition, we have begun to incorporate the expression data for 30 other cancer types from the TCGA database (Supplementary Table S2). A total of 65 cell line datasets representing 23 of these cancer types have also been included (Supplementary Table S3). The full list of the datasets can be found in the ‘Data Source’ panel of KM-Express.

**Table 1.** Datasets for human prostate cancer patients and cell lines

Accession ID	PMID	Phenotype	Sample size
TCGA		Tumor	384
SRP011439	21 804 560	Benign vs localized tumor	114
SRP005908	21 036 922	Prostate patients	28
GSE24283	21 261 984	Tumor vs normal	3/3
SRP002628	21 571 633	Tumors and adjacent normal tissues	20/10
ERP000550	22 349 460	Tumor and adjacent normal tissues	14/14
SRP026387	24 054 872	Pre and post treatment	7/7
GSE48812	25 044 704	PrEC, LNCaP, PC3	12/12/12
SRP013621	24 012 641	Tumor	1/1
SRP027258	26 805 894	PC3, LNCaP, PrEC	12/12/12
SRP010054	25 700 553	LNCaP	3/3
SRP014759	25 274 489	Tumor	1/1
GSE25183	21 804 560	Normal vs tumor	58

### Functionality

KM-Express provides a simple user-friendly web interface that enables users to perform multiple analyses including survival analysis based on patient RNA-seq datasets and gene expression analysis based on patient and cell line RNA-seq datasets. In addition, KM-Express provides a module for statistical tests to assess the differential significance of the selected gene(s). KM-Express is very simple to use and analyses can be accomplished in about one minute. The results from KM-Express are provided as high-resolution PNG format or they can be download as PDFs if users want to make modifications on them.

For a given candidate gene, KM-Express can:

- Determine if it is potential prognostic markers or not. According to the survival analysis results, users can infer its potency to predict patient outcome.
- Check if it is an oncogene or a tumor suppressor by comparing its expression level between normal and tumor samples. Multiple independent datasets from different labs enable a cross-validation of the gene to give more confidence to its role as an oncogene or a tumor suppressor.
- Help in the design of wet lab experiments. Users can refer to the gene expression level in different cell lines or under different interventions or manipulations, which facilitates the hypothesis generation on gene function.

#### Survival analysis

**Figure 2.**
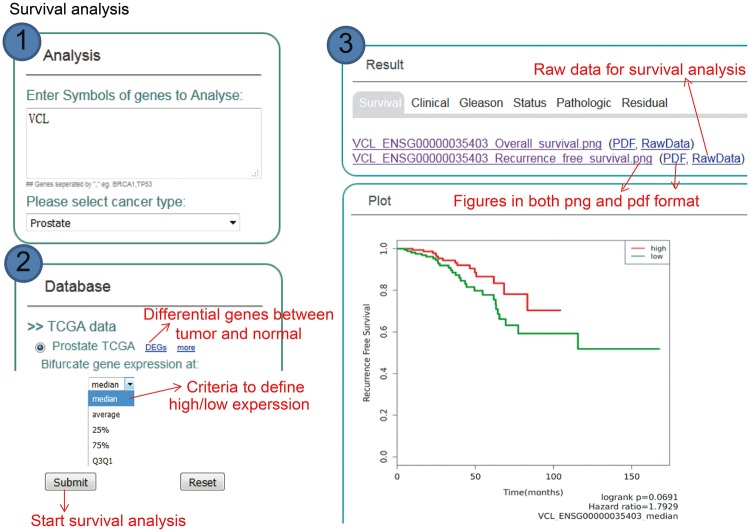
Step-by-step usage of KM-Express for survival analysis. (1) Enter the gene symbol in the box provided. When users input multiple genes, gene symbols should be separated by ‘,’. The expression level of the multiple genes will be averaged and used for survival analysis. After the cancer type is selected, the RNA-seq data available for survival analysis will be shown in ‘Database’ panel. (2) Select the survival measure. The survival outcome is calculated as biochemical recurrence for tumor or without tumor for prostate cancer and dead or alive status for breast cancer. Next, select the criteria to divide the patients into two groups, one group highly expressing the input gene, the other group lowly expressing the input gene. For median/average/25%/75% cutoff, higher than the threshold corresponds to highly express group, others are lowly express group. For Q3Q1, highly expressed group represents patients with input gene expression >75% patients, while the lowly expressed group represents patients with input gene expression <25% patients. (3) The output provides KM-plot figures in both PDF and PNG format. The significance *P*-value and hazard ratio are provided in the figure along with the summary of input genes and user-selected parameters.

One major function of KM-Express is patient survival analysis (Figure 2). To perform survival analysis, users simply go to the ‘Analysis’ panel of the homepage and type in the gene symbol of the gene(s) they are interested in (separated by a comma if more than one gene) and then select the cancer type they want to look at (e.g. prostate or breast cancer). Users then choose the survival measure and expression cutoff to divide patients into two groups. After users submit the survival job, a Kaplan–Meier plot will show if there is a significant difference in survival time between the two groups of patients. The output figures are provided to users as publication quality high-resolution PNG and PDF formats. If users prefer to reproduce or perform survival analysis using their own personalized criteria, they can download the raw data from the ‘RawData’ tab of the output page. If the TCGA dataset is selected, gene expression analysis is simultaneously performed with survival analysis.

#### Gene expression analysis

**Figure 3.**
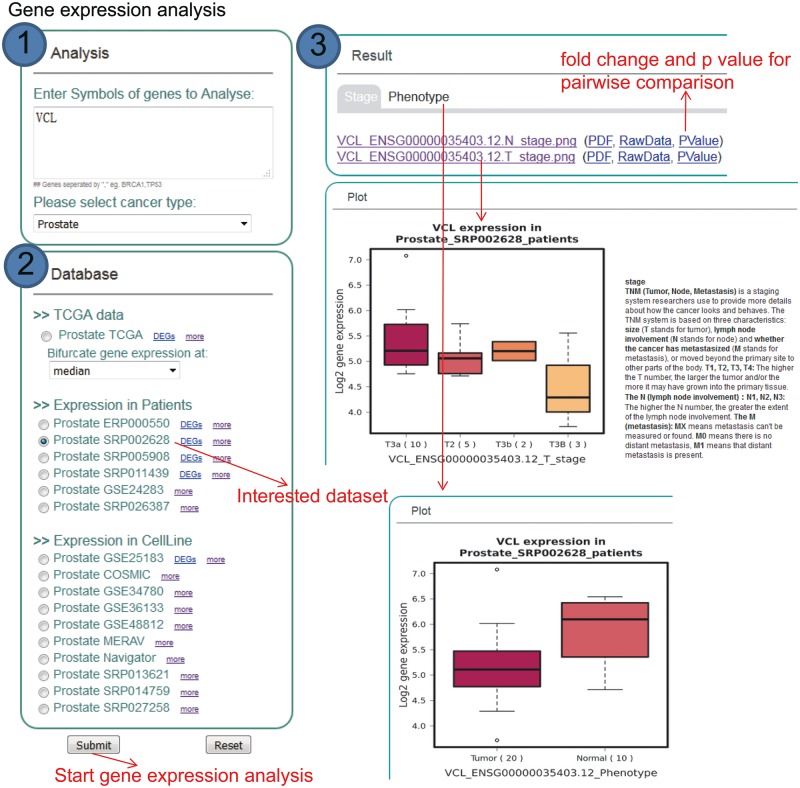
Step-by-step usage of KM-Express for expression analyses. (1) Enter the gene symbol in the box provided. When users input multiple genes, gene symbols should be separated by ‘,’. After selecting the cancer type, the RNA-seq data available for expression analysis will be shown in ‘Database’ panel. (2) Select a dataset to compare the expression of input genes in different groups of patients/cell lines, according to clinical, pathological status of patients or time-series drug treatment or different cell line types. (3) The output provides expression level of input genes in different groups of patients according to their clinical, pathological status, time-series drug treatment or different cell line types.

KM-Express is also designed to perform gene expression analysis based on patients and cell line datasets (Figure 3). In the ‘Analysis’ panel of the homepage, users type in the gene symbol of the gene(s) they are interested in and then select the cancer type and a dataset from either patients or cell lines. After the job has been submitted and the analysis is finished, the result of the expression comparison for the input gene will be shown as a boxplot and will be categorized according to the clinical or pathological information based on the study. To help users better understand the clinical or pathological terms, interpretive statements are shown along with the boxplot.

We realize that not all potential KM-Express users will already have candidate biomarker gene(s) and thus may want to identify their own using the datasets archived in KM-Express. To facilitate this, we have included in KM-Express an option where users can obtain differentially expressed genes (DEGs) via the tab ‘DEGs’ (Figures 2 and 3) on any of the patient datasets that we currently have in KM-Express. Currently, KM-Express will provide a list of DEGs between normal and tumor samples with a fold change >1.2 and *P* < 0.05 (note: KM-Express will only do this for datasets that contain >3 tumors and 3 normal samples). Once users find the candidate gene(s) they are interested in, they can then perform similar survival and gene expression analysis as described above.

#### Statistical tests for differential significance

**Figure 4.**
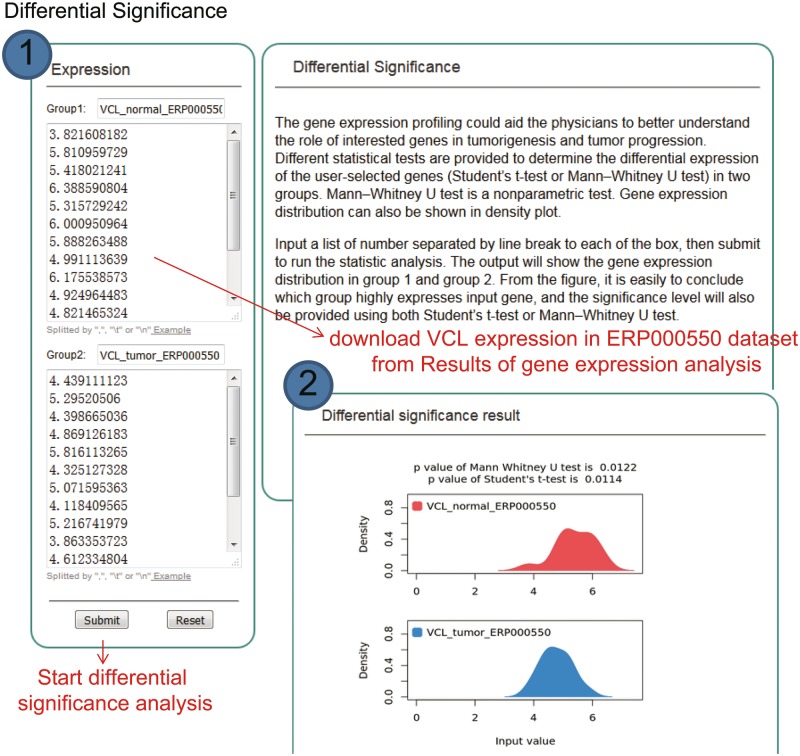
Step-by-step usage of KM-Express for differential significance test. (1) Input the gene expression level in two groups, separating each number by a line break, tab or comma, and then submit to run the statistics analysis. Two statistical tests are simultaneously provided to determine the differential significance of the user-selected genes (Student’s *t*-test and Mann–Whitney *U*-test) in two groups. (2) The output provides a gene expression distribution density plot. The significance level is also provided using both Student’s *t*-test and Mann–Whitney *U*-test.

KM-Express provides statistical tests to determine whether a gene is significantly differentially expressed between any two groups. Since a dataset can sometime contain many different cancer types or conditions (e.g. such as breast cancer) and therefore many comparisons, we have provided a downloadable file under the ‘PValue’ tab (Figure 3, under Results) which contains both the fold change and the corresponding *P*-value for each pairwise comparison. If users would like to examine the expression of their gene(s) in any two clinical or pathological subgroups in more detail, they can download the raw expression data from the ‘RawData’ tab (Figure 3, under Results) and perform their own statistical tests or use the ‘Differential Significance’ function in KM-Express (Figure 4). As shown in Figure 4, users input the expression values of the gene into two groups (e.g. Group 1 = normal and Group 2 = tumor). After the statistical test is complete, a density plot of the gene expression distribution for each group is shown along with the significance using the Student’s *t*-test and the Mann–Whitney *U*-test.

While the ‘Differential Significance’ function was designed for analyzing data from KM-Express, we believe it can also serve as a useful tool for users if they wish to examine the statistical significance of the expression difference for their target genes from their own in-house data.

#### Data maintenance and update

We have implemented a function panel ‘DataSubmit’ that enables users or maintainers of KM-Express to submit new datasets by following the format of the example files. The datasets in KM-Express will be updated approximately every 6 months.

#### Documentation, tutorial and help

Documentation for KM-Express includes data source, help and detailed tutorials (http://ec2-52-201-246-161.compute-1.amazonaws.com/kmexpress/Tutorial.php). In the ‘Data Source’ page, a detailed description (Table 1 and Supplementary Tables S1, S2 and S3) is provided for each dataset including the accession ID, publication, sample size, normal/tumor/metastatic phenotype and clinical/pathological characteristic. A step-by-step tutorial is provided for users to quickly execute examples using KM-Express. The ‘Help’ page allows users to have a better understanding of the clinical or pathological terms, which are used to illustrate the cancer staging.

## Validation and application

**Figure 5.**
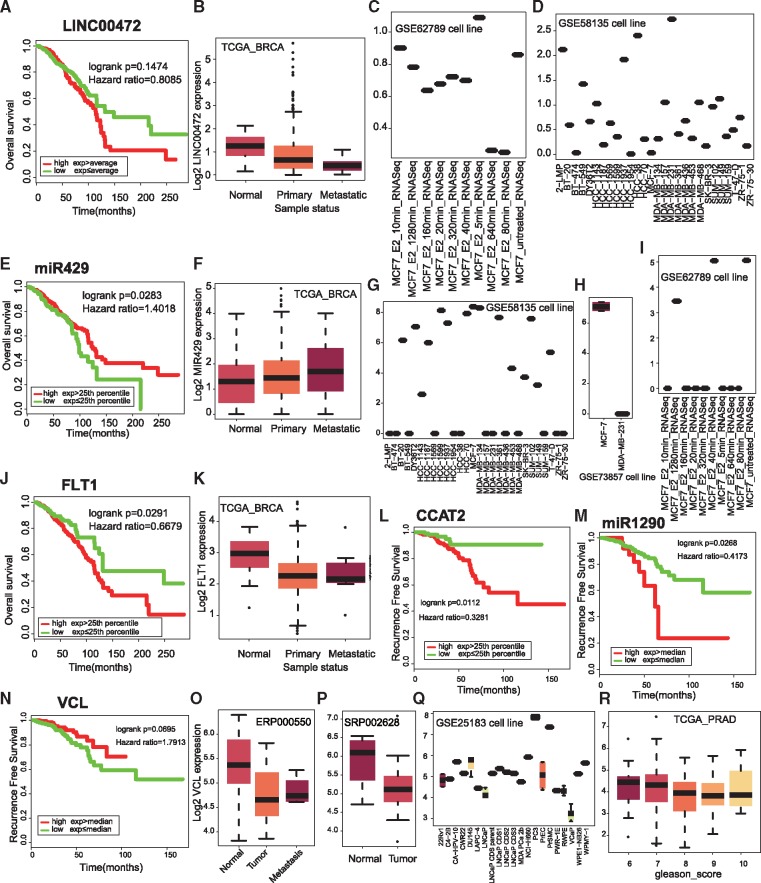
Validation of previously reported prognostic biomarkers. (**A**) Kaplan–Meier plot for lncRNA LINC00472. (**B**) Differential expression of LINC00472 among tumor and normal patients. Expression change of LINC00472 after estradiol (E2) treatment in MCF7 cells (**C**) and in 28 breast cancer cell lines (**D**). (**E**) Kaplan–Meier plot for miR429. (**F**) Differential expression of miR429 in metastatic patients compared with primary breast cancer patients and normal tissue. Expression of miR429 in different cell lines (**G, H**) and after E2 treatment (**I**). (**J**) Kaplan–Meier plot for the protein coding gene VEGFR1 (FLT1). Expression of FLT in normal breast samples and cancer patients (**K**). Kaplan–Meier plot for lncRNA CCAT2 (**L**) and miR1290 (**M**) and VCL (**N**). Differential expression of VCL between normal and tumor prostate (**O, P**) and in prostate cancer cell lines (**Q**). (**R**) VCL is differentially expressed in patients with different Gleason score.

To demonstrate the usefulness of KM-Express on biomarker discovery and functional validation, we examined several previously reported RNA biomarkers for breast cancer including the long non-coding RNA (lncRNA) LINC00472 (29), miR429 (30, 31) and VEGFR1 (FLT1) (32). In general, the results from KM-Express for patient survival and gene expression data in normal vs tumor samples were consistent with previous findings for the three RNA biomarkers (Figure 5A, B, E, F, J and K). The only inconsistent result was for the patient survival finding of LINC00742, which could be attributed to the larger and more diverse cohorts included in the TCGA dataset. In addition to breast cancer biomarkers, we also examined RNA biomarkers for prostate cancer such as CCAT2 (33), miRNA1290 (34) and VCL (35). Again, KM-Express was able to obtain similar results as previously reported (Figure 5L–P).

A unique and useful feature which has been incorporated into KM-Express and is not found in other biomarker discovery tools is the inclusion of cell line gene expression information. We have added this data in KM-Express so that biologists can have the ability in the same tool to examine the gene expression profile of their RNA biomarkers not only in patient samples but also in different cell lines. This feature will negate the need to find and use other tools and thus will facilitate the design of downstream functional assays to test whether RNA biomarkers are also potential therapeutic targets.

This functionality is nicely illustrated for the breast cancer biomarkers LINC00472 and miR429 (Figure 5). Specifically, the cell line gene expression data for LINC00472 in KM-Express shows that the transcript is expressed only in MCF-7 cells after estrogen stimulation (Figure 5C) and its expression level is the highest in MDA-MB-231 cells among 28 breast cancer cell lines (Figure 5D). In contrast, KM-Express shows miR429 expression level is the highest in MCF-7 cells (but lowest in MDA-MB-231 cells) and the level of the miRNA is dramatically reduced after estrogen stimulation (Figure 5I). The cell line information for the two biomarkers would therefore suggest that MCF-7 is a good cell line model for studying the function and hormone regulation of both transcripts while MDA-MB-231 cells would make a good cell line model for LINC00472 knock-down studies and miR429 overexpressing work.

## Future extensions

We will manually curate newly generated RNA-seq datasets for patients or cell lines every 6 months. Currently, users can perform both expression and survival analysis with breast and prostate cancers on KM-Express. Users can also carry out similar analysis on 30 other cancer types based on TCGA data with cell line expression analysis for most of the cancer types. We will continue to expand and include new datasets for all these cancer types.

## Discussion

We have developed KM-Express, a simple user-friendly web-based tool. KM-Express is not the only web-tool which can identify prognostic markers based on RNA-seq transcriptome dataset, however, it is different from other web-tools in that it, (i) contains NGS transcriptome data in addition to TCGA datasets, (ii) provides detailed expression comparison of interested genes in cancer compared to normal, or in different subgroups of patients according to the molecular, genetic, clinical and pathological status, (iii) helps the researcher in experimental design based on the expression of gene(s) in different cell lines and (iv) includes an analysis tool to visualize and statistically determine the significance of gene-centric expression differences in two groups. To ensure quick and easy searching ability, an intuitive querying interface was implemented. The survival analysis is very quick and is accomplished in about one second.

In the coming years, expression data based on NGS will continue to accumulate for many cancers, highlighting the importance of a published web-tool providing comprehensive information about prognostic markers, cross-dataset expression and experimental support from cell lines.

## Conclusions

In brief, KM-Express hosts comprehensive NGS-based expression profiles and provides convenient and user-friendly tools to explore potential prognostic markers. We have generated KM-Express as a hypothesis generation tool for researchers to identify potential prognostic RNA biomarkers to follow-up for further research. KM-Express will help accelerate translational research from bench to bedside.

## Supplementary data

Supplementary data are available at *Database* Online.

## Supplementary Material

Supplementary Data
